# ASF1 is required to load histones on the HIRA complex in preparation of paternal chromatin assembly at fertilization

**DOI:** 10.1186/s13072-018-0189-x

**Published:** 2018-05-11

**Authors:** Béatrice Horard, Laure Sapey-Triomphe, Emilie Bonnefoy, Benjamin Loppin

**Affiliations:** Laboratoire de Biométrie et Biologie Evolutive - CNRS - UMR5558, Université Claude Bernard Lyon I, 16, rue R. Dubois - Bât. G. Mendel, 69622 Villeurbanne Cedex, France

**Keywords:** ASF1, Histone chaperone, Drosophila, Early embryo, Pronucleus, Haploid, Chromatin assembly

## Abstract

**Background:**

Anti-Silencing Factor 1 (ASF1) is a conserved H3–H4 histone chaperone involved in both Replication-Coupled and Replication-Independent (RI) nucleosome assembly pathways. At DNA replication forks, ASF1 plays an important role in regulating the supply of H3.1/2 and H4 to the CAF-1 chromatin assembly complex. ASF1 also provides H3.3–H4 dimers to HIRA and DAXX chaperones for RI nucleosome assembly. The early *Drosophila* embryo is an attractive system to study chromatin assembly in a developmental context. The formation of a diploid zygote begins with the unique, genome-wide RI assembly of paternal chromatin following sperm protamine eviction. Then, within the same cytoplasm, syncytial embryonic nuclei undergo a series of rapid, synchronous S and M phases to form the blastoderm embryo. Here, we have investigated the implication of ASF1 in these two distinct assembly processes.

**Results:**

We show that depletion of the maternal pool of ASF1 with a specific shRNA induces a fully penetrant, maternal effect embryo lethal phenotype. Unexpectedly, despite the depletion of ASF1 protein to undetectable levels, we show that *asf1* knocked-down (KD) embryos can develop to various stages, thus demonstrating that ASF1 is not absolutely required for the amplification of cleavage nuclei. Remarkably, we found that ASF1 is required for the formation of the male pronucleus, although ASF1 protein does not reside in the decondensing sperm nucleus. In *asf1* KD embryos, HIRA localizes to the male nucleus but is only capable of limited and insufficient chromatin assembly. Finally, we show that the conserved HIRA B domain, which is involved in ASF1-HIRA interaction, is dispensable for female fertility.

**Conclusions:**

We conclude that ASF1 is critically required to load H3.3–H4 dimers on the HIRA complex prior to histone deposition on paternal DNA. This separation of tasks could optimize the rapid assembly of paternal chromatin within the gigantic volume of the egg cell. In contrast, ASF1 is surprisingly dispensable for the amplification of cleavage nuclei, although chromatin integrity is likely compromised in KD embryos.

**Electronic supplementary material:**

The online version of this article (10.1186/s13072-018-0189-x) contains supplementary material, which is available to authorized users.

## Background

The controlled accessibility of DNA to a variety of proteins is essential for the functioning of eukaryotic genomes. Basic nuclear activities such as gene transcription, DNA repair or DNA replication all require the disassembly of nucleosomes to allow the appropriate protein complexes to access specific DNA sequences or regions. However, these dynamic transactions must be compensated by the reassembly of chromatin over nucleosome-depleted regions in order to maintain chromatin integrity. Nucleosome assembly begins with the association of two histone H3–H4 dimers to form a (H3–H4)_2_ tetramer on DNA. The particle is then completed with the addition of two H2A-H2B dimers to form an octameric nucleosome [1, 2]. From their synthesis in the cytoplasm to their final deposition on DNA, histones are constantly associated with chaperones [3]. Histone chaperones form a heterogeneous group of proteins with various functions, such as storage, transport, modification or deposition of histones on DNA [4, 5]. Originally discovered in the budding yeast, Anti-Silencing Factor 1 (ASF1) is a small, conserved histone chaperone of the H3/H4 family that is involved in a variety of chromatin-related functions, such as nucleosome assembly and disassembly, chromatin remodeling, gene silencing or DNA damage checkpoint [6–9]. This diversity of functions reflects the implication of ASF1 in both Replication-Coupled (RC) and Replication-Independent (RI) histone deposition pathways. Studies in the budding yeast and metazoan have indeed established that ASF1 interacts with subunits of the Cac/CAF-1 (Chromatin Assembly Factor 1; RC) and Hir/HIRA (Histone Regulator-A; RI) complexes, respectively [10–14]. Structural studies have revealed that ASF1 interacts with H3 and H4 as dimers by binding the so-called tetramerization interface. This likely regulates the initiation of nucleosome assembly by preventing uncontrolled formation of heterotetramers [3, 9, 14–16]. During DNA replication in animal cells, ASF1 plays a pivotal role in controlling the flux of H3–H4 dimers to the CAF-1 complex, in close cooperation with the replicative helicase subunit MCM2 [17, 18]. Notably, ASF1 knock-down (KD) in *Drosophila* and human cells blocks the progression of DNA replication forks [8, 17]. On the other hand, the functional relationship between ASF1 and the HIRA complex has been essentially characterized in the context of transcriptional regulation. For instance, HIRA and ASF1 cooperate for the transcriptional activation of Mef2 target genes in a cell model of mouse muscle differentiation [19]. During the activation of *hsp70* (heat-shock protein 70) genes on *Drosophila* polytene chromosomes, ASF1 presumably cooperates with HIRA and the dATRX/XNP chromatin remodeler to fill nucleosome gaps at these loci [20]. HIRA, UBN1 and ASF1a associate at different regulatory elements over the human genome to allow H3.3 deposition, including active or poised enhancers as well as the transcription start sites of highly transcribed genes [21]. Finally, ASF1 and HIRA cooperate for the transcriptional silencing of heterochromatin regions in fission yeast [22] and the formation of senescence-associated heterochromatin foci in human cells [13].

In *Drosophila* and mammals, the HIRA complex is critically required for the de novo assembly of nucleosomes on the paternal pronucleus at fertilization [23–25]. Paternal chromatin assembly is a genome-wide, RI assembly process, which begins concomitantly with the eviction of sperm nuclear basic proteins (SNBPs) [26]. We previously reported that both subunits of the *Drosophila* HIRA core complex (HIRA and YEM, the fly ortholog of mammalian UBN1) specifically localize in the decondensing sperm nucleus where they allow deposition of H3.3–H4 histone dimers on paternal DNA [23, 27, 28]. (H3.3–H4)_2_ tetramers must be subsequently complemented with either H2A-H2B or H2A.Z-H2B histone dimers to finalize the nucleosome assembly process [29]. In this context, we previously reported the unexpected observation that, in contrast to HIRA and YEM, ASF1 was apparently absent from the male pronucleus during nuclear decondensation, thus questioning its role, if any, in this RI assembly process [27]. In addition, during the syncytial phase of embryonic development, ASF1 is known to accumulate in nuclei at each S phase, but its actual requirement for the rapid amplification of embryonic nuclei has not been investigated. In this study, we have functionally addressed the respective contributions of ASF1 in these two distinct nucleosome assembly pathways during early embryo development.

## Results

### Maternal ASF1 is essential for embryo development

**Fig. 1:**
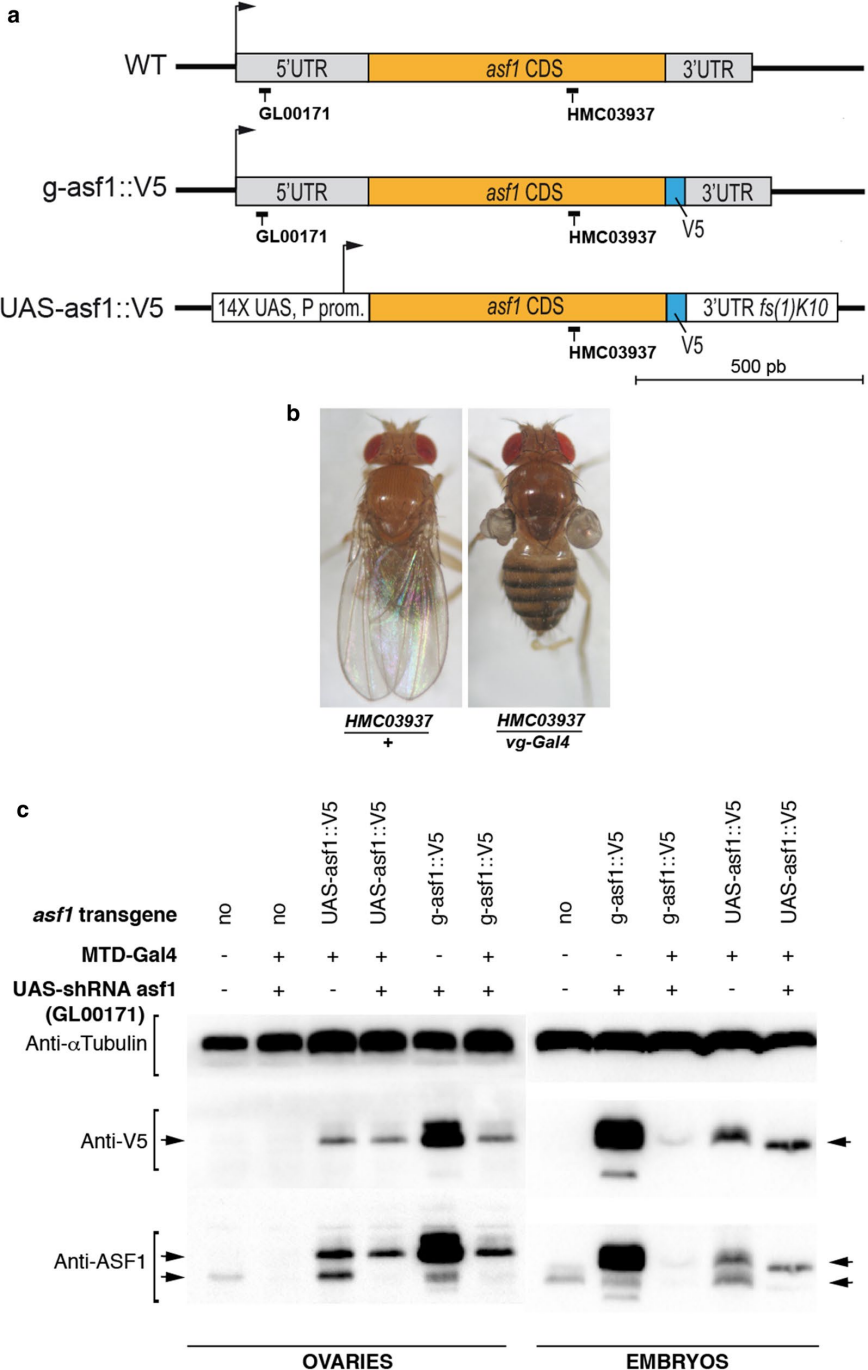
*asf1* knock-down and rescue experiments. **a** Schemes of the *D. melanogaster asf1* gene and rescue transgenes used in this study. The respective target sites of the GL00171 and HMC03937 shRNAs are indicated. **b** Left: a control *P[TRiP.HMC03937]/*+ adult showing normal wings. Right: induction of the HMC03937 shRNA in the wing imaginal disk with *vg-Gal4* results in severe wing atrophy in all tested flies (*n* = 224). **c** Western blot analysis of endogenous or V5-tagged ASF1 extracted from ovaries or embryos of indicated genotypes. The anti alpha-Tubulin immunostaining was used as a loading control. Note that the *UAS*-*V5*-*asf1* transgene does not contain the 21-bp target of the *asf1* GL00171 shRNA. The anti-ASF1 antibody recognizes both wild-type and tagged versions of ASF1. Arrows indicate specific bands

In contrast to most eukaryotes where two *asf1* paralogs (*asf1a* and *asf1b*) are commonly found, *Drosophila melanogaster* has a single *asf1* gene. Hypomorphic *asf1* alleles are zygotic lethal at the embryonic or larval stages [30]. To address the function of maternal ASF1, we chose to specifically knock-down *asf1* expression in adult female germ cells using transgenic small hairpin RNAs (shRNAs) whose expression can be induced with germline-specific GAL4 drivers [31]. The first tested shRNA (HMC03937), which is expressed from a pValium20 vector with a minimal *hsp70* promoter [31], targets a sequence of 21 nucleotides within the coding region of *asf1* (Fig. 1a). We observed that induction of HMC03937 with the ubiquitous *Act5C*-*Gal4* driver was zygotic lethal (all F1 adults had the *CyO* balancer chromosome from the *Act5C*-*Gal4/CyO* parent; *n* = 613). In addition, its specific induction in wing imaginal disks with the *vestigial (vg)*-*Gal4* driver efficiently prevented wing development (100%; *n* = 224) suggesting that *asf1* is required for cell proliferation in somatic tissues (Fig. 1b). We then turned to another shRNA (GL00171), which targets the predicted 5′UTR of *asf1* (Fig. 1a). This shRNA is expressed from the pValium22 vector that carries a germline-specific promoter and 3′UTR [31]. Accordingly, GL00171 did not cause any somatic phenotype when combined with either *Act5C*-*Gal4* (viable) or *vg*-*Gal4* (normal wing development). Interestingly, however, although oogenesis appeared mostly unaffected by germline expression of GL00171 (using either “MTD-Gal4” or *nos*-*Gal4* drivers), eggs laid by KD females never hatched (Table 1). To verify that this maternal effect, embryonic lethal phenotype was indeed caused by the silencing of *asf1*, we designed a Gal4 inducible, *UASP*-*asf1::V5* rescue transgene that lacks the GL00171 target sequence (Fig. 1a). This transgene fully rescued the fertility of KD females (Table 1). In contrast, a transgene expressing ASF1::V5 under its endogenous upstream regulatory region (*g*-*asf1::V5*; Fig. 1a) poorly rescued the *asf1* KD phenotype when females were raised at 25 °C and had no rescuing effect at 29 °C (Table 1). Western blot analyses confirmed the severe reduction of ASF1 protein levels in KD ovaries or in embryos produced by KD females (hereafter KD embryos) (Fig. 1c). It also revealed that the *g*-*asf1*-*V5* transgene produced massive amounts of ASF1::V5, likely because of a duplication event at the insertion site (not shown). Still, the level of ASF1::V5 produced by this transgene was drastically reduced in KD ovaries and barely detectable in KD embryos, thus confirming the high silencing efficiency of the GL00171 shRNA (Fig. 1c).

**Table 1:** Fertility tests

Females	Temperature (°C)	Males	Number of eggs	Hatching rate (%)
*MTD*-*Gal4 *> *UAS*-*shRNA asf1*	25	*w* ^*1118*^	1715	0
*nos*-*Gal4 *> *UAS*-*shRNA asf1*	25	*w* ^*1118*^	987	0
*MTD*-*Gal4 *> *UAS*-*shRNA asf1*	25	*g*-*cid*-*EGFP::cid*	276	0
*MTD*-*Gal4 *> *UAS*-*shRNA Hira*	25	*g*-*cid*-*EGFP::cid*	354	0
*MTD*-*Gal4 *> *UAS*-*shRNA yem*	25	*g*-*cid*-*EGFP::cid*	489	0
*UAS*-*asf1::V5(62E), UAS*-*shRNA asf1/TM6*	25	*w* ^*1118*^	295	91.07
*MTD*-*Gal4 *> *UAS*-*asf1::V5(62E), UAS*-*shRNA asf1*	25	*w* ^*1118*^	674	92.41
*MTD*-*Gal4 *> *UAS*-*asf1::V5(62E), UAS*-*shRNA asf1*	29	*w* ^*1118*^	462	80.97
*g*-*asf1::V5(62E), asf1*^*2*^*/asf1*^*1*^	25	*g*-*asf1::V5(62E), asf1*^*2*^*/asf1*^*1*^	172	66.2
*g*-*asf1::V5(62E), asf1*^*2*^*/asf1*^*1*^	25	*w* ^*1118*^	40	67.2
*g*-*asf1::V5(62E), UAS*-*shRNA asf1*/TM6B	25	*w* ^*1118*^	349	78.7
*g*-*asf1::V5(62E), UAS*-*shRNA asf1*	25	*w* ^*1118*^	530	74.9
*g*-*asf1::V5(62E), MTD*-*Gal4* > *UAS*-*shRNA asf1*	25	*w* ^*1118*^	1891	5.3
*g*-*asf1::V5(62E), MTD*-*Gal4 *> *UAS*-*shRNA asf1*	29	*w* ^*1118*^	1314	0

**Fig. 2:**
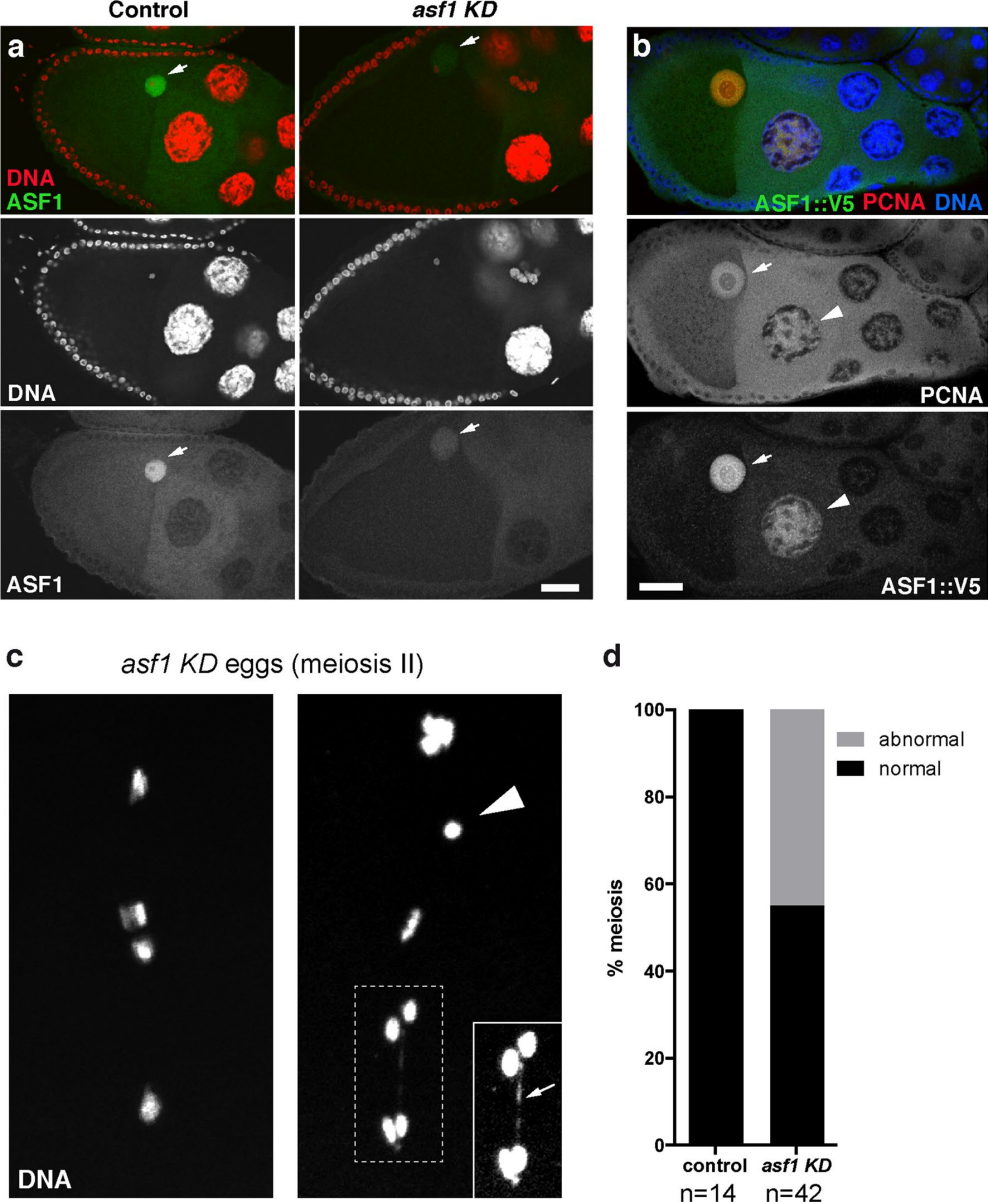
*asf1* KD does not affect oogenesis but frequently impairs meiotic divisions. **a** Stage 9 egg chambers from control *w*^*1118*^ (left) and *asf1* KD (right) ovaries stained for ASF1 (green) and DNA (red). In control egg chambers, ASF1 accumulates in the germinal vesicle (arrows; *n* = 30). In ovaries expressing the *asf1* shRNA in germ cells, ASF1 is still weakly detected in the oocyte nucleus (arrows) at the same stage (*n* = 14). Scale bar: 20 μm. n indicates the number of egg chambers analyzed. **b** A stage 9 egg chamber expressing V5-tagged ASF1 protein (green) from a genomic *asf1*-*V5* transgene (*g*-*asf1::V5*). The ASF1::V5 protein accumulates in the germinal vesicle (arrow) but is also enriched in nurse cell nuclei (arrowhead) positive for the replication factor PCNA (red; *n* = 12). DNA is in blue. Scale bar: 20 μm. Color panels in a and b show merged stainings. **c** Chromosomal defects are frequently observed during the second meiotic division in *asf1* KD eggs. The meiosis in the left panel appears normal but the one on the right shows a lagging chromosome (arrowhead) and a chromatin bridge (arrow). The chromatin bridge is more visible in the inset where the contrast has been intentionally increased. Scale bar: 20 μm. **d** Quantification of abnormal female meiotic divisions in *asf1* KD eggs

Using immunofluorescence, we nevertheless observed the persistence of low levels of ASF1 protein in KD ovaries, thus providing a plausible explanation for the absence of obvious oogenesis defects in KD females. In stage 8-10 egg chambers of control females, ASF1 accumulated in the nucleoplasm of the germinal vesicle (GV), *i.e.* the oocyte nucleus. This staining was greatly reduced in egg chambers from KD ovaries, but not completely abolished (Fig. 2a). The same distribution was observed using anti-V5 antibodies to detect the tagged version of ASF1. ASF1::V5 was also occasionally detected in individual nurse cell nuclei that were positive for the DNA replication marker PCNA (Fig. 2b) consistent with its implication in RC chromatin assembly [8].

Cytological observations of eggs from KD females revealed frequent chromosome segregation defects during the second meiotic divisions (Fig. 2c, d). Although this indicated a role of ASF1 in the maintenance of oocyte chromosome integrity, the incomplete penetrance of this phenotype implied the existence of additional defects that could account for the fact that *asf1* KD embryos never hatched.

### Efficient depletion of maternal ASF1 does not abolish nuclear divisions in early embryos

**Fig. 3:**
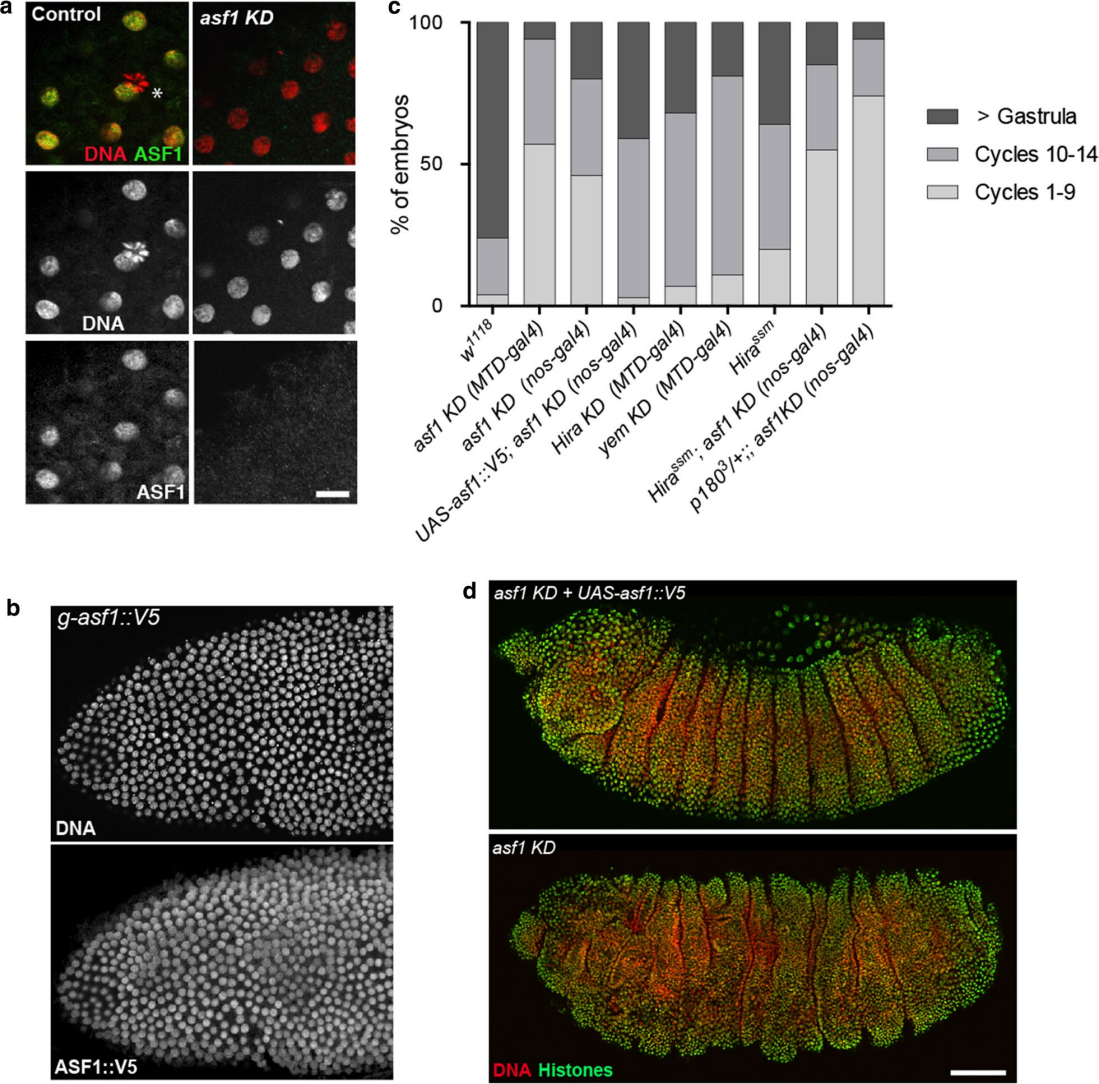
Depletion of maternal ASF1 does not prevent embryo development. **a** In control early syncytial embryos (left panels), ASF1 accumulates in nuclei at each synchronous S phase (*n* = 24). Note that ASF1 is not detected in the rosette of condensed chromosomes that form the polar body (asterisk). In embryos from *asf1* KD females (right panels), nuclear amplification is observed despite the apparent absence of ASF1 (*n* = 28). Scale bar: 10 μm. **b** A syncytial blastoderm embryo showing the accumulation of ASF1::V5 in replicating nuclei. ASF1::V5 is expressed from a *g*-*asf1::V5* transgene and is detected with an anti-V5 antibody. **c** Quantification of embryonic phenotypes corresponding to the indicated genotypes. Embryos were collected every 2 h and then allowed to develop for 2 h at 25 °C before fixation and developmental stage determination. A minimum of 150 embryos were scored for each genotype. Corresponding raw data are shown in Additional file 3: Table S1. **d** *asf1* KD rescue with a UAS-asf1::V5 transgene. Top panel: A representative stage 13 embryo from a *asf1* KD female expressing the rescue transgene *UAS*-*asf1::V5* (*n* = 62). Lower panel: A representative late *asf1* KD embryo at approximately the same stage showing aberrant segmentation (*n* = 57). Color panels in a and d show merged stainings. Scale bar: 50 μm

As previously reported, during the rapid embryonic syncytial divisions, maternal ASF1 accumulated in cleavage nuclei at each S phase but remained in the cytoplasm during mitoses [27, 30], in a way similar to DNA replication factors [32, 33] (Fig. 3a). We also confirmed that ASF1::V5 detected with anti-V5 antibodies also enters replicating nuclei at each S phase, consistent with its role in S phase progression (Fig. 3b). Interestingly, ASF1 was never detected in replicating/interphasic nuclei of *asf1* KD embryos (100%, *n* = 28; Fig. 3a), indicating that the residual pool of ASF1 present in the germinal vesicle of KD oocytes does not contribute detectable levels of the protein to the egg. The observation of KD embryos that reach the blastoderm stage without detectable level of ASF1 thus demonstrates that this histone chaperone is not absolutely required for the amplification of embryonic cleavage nuclei (Additional file 1: Fig. S1). We nevertheless observed that most *asf1* KD embryos showed abnormal mitoses, with chromatin bridges frequently connecting dividing nuclei. Developing *asf1* KD embryos accumulated aneuploid nuclei that appeared to divide asynchronously (Additional file 2: Fig. S2**)**. These abnormal divisions account for the high proportion of *asf1* KD embryos that arrest development before nuclear cycle 9 (Fig. 3c and Additional file 3: Table S1). Finally, the minority of *asf1* KD embryos that developed beyond gastrulation all showed highly irregular segmentation compared to viable, *UASP*-*asf1*-*V5* rescued embryos (Fig. 3d).

### ASF1 is required for HIRA-dependent assembly of paternal chromatin

**Fig. 4:**
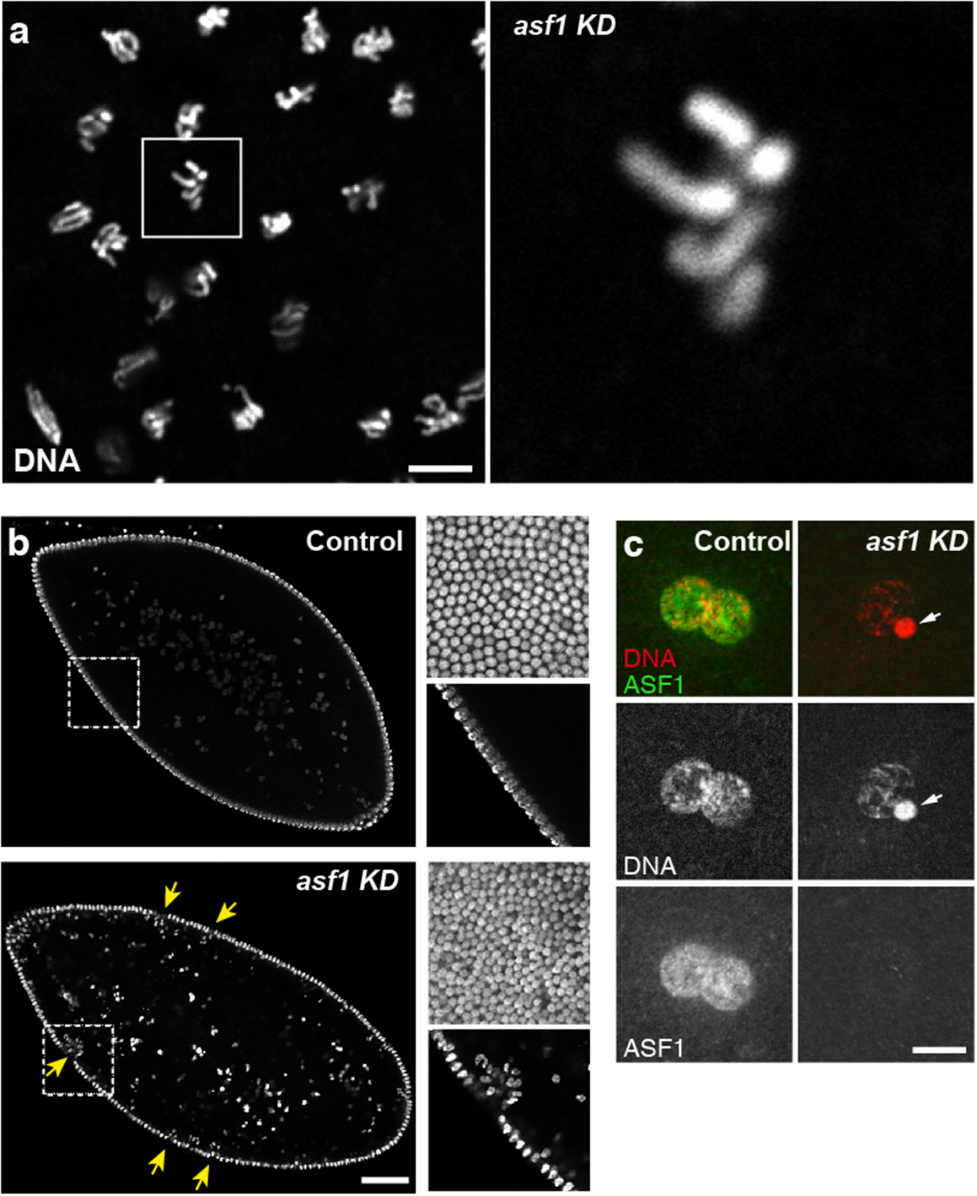
Gynohaploid development of *asf1* KD embryos. **a** A group of syncytial nuclei in metaphase from an *asf1* KD embryo. These nuclei are haploid (four chromosomes), as visible in the close-up. Scale bar: 10 μm. **b** Confocal sections of cellularized *asf1* KD (*n* = 67) and control blastoderm (*n* = 83) embryos. Insets were taken at the surface of the same embryo (top) or in the same plane than the main image (bottom). *asf1* KD embryos typically show irregular organization of the nuclear layer, with frequent patches of nuclear sinking (yellow arrows). Note the doubled nuclear density at the surface in haploids compared to the diploid control. Scale bar: 50 μm. **c** In control eggs (left) at pronuclear apposition, ASF1 (green) is abundant in both apposed pronuclei (100%; *n* = 20). In *asf1* KD eggs (right) at the same stage, no signal is detected in apposed pronuclei (0%; *n* = 24). Note that the female pronucleus appears normal while the male pronucleus fails to decondense (arrowhead). Scale bar: 10 μm. Color panels in c show merged stainings

**Fig. 5:**
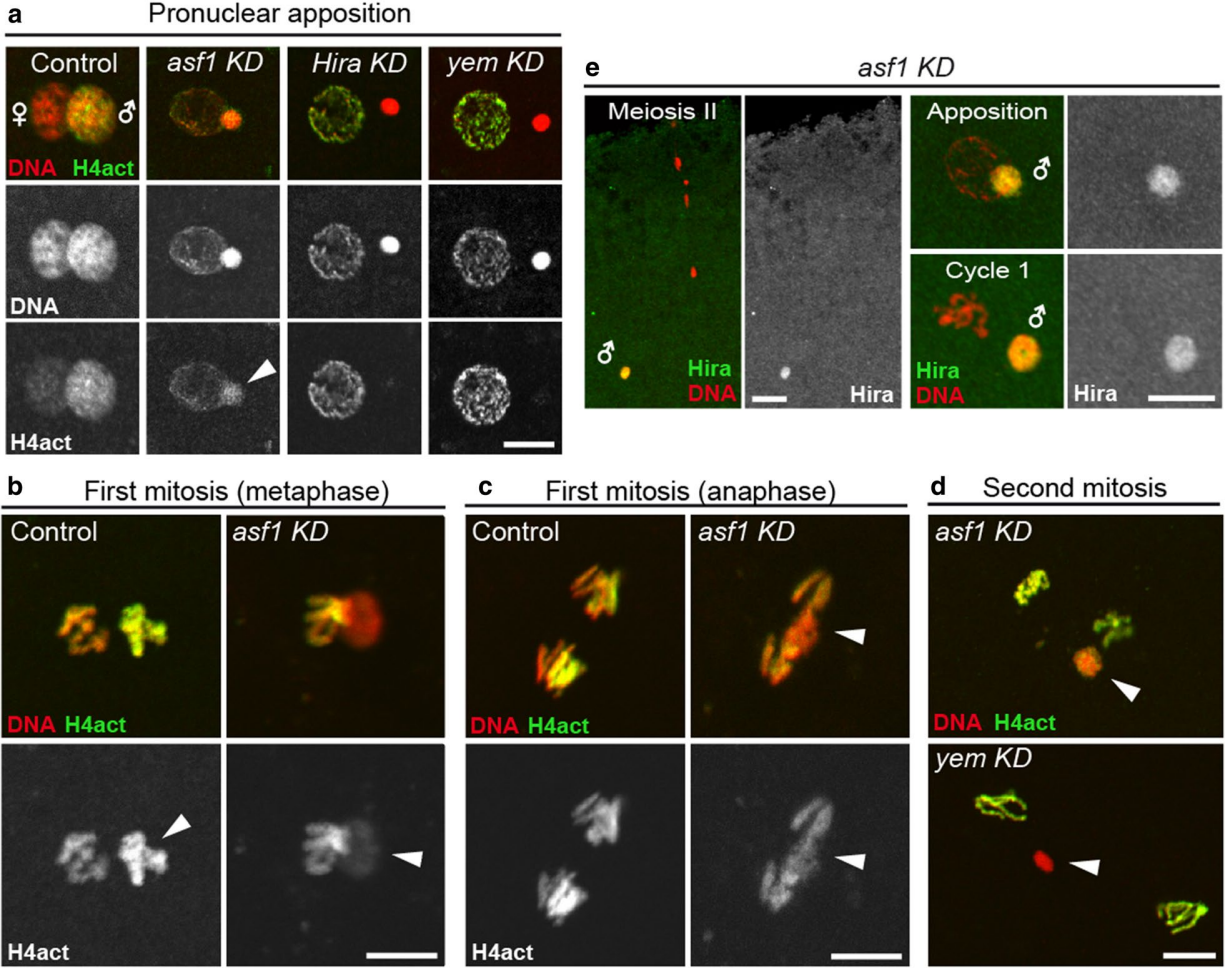
ASF1 is required for HIRA-dependent assembly of paternal chromatin. **a** Confocal images of eggs at pronuclear apposition stained for acetylated histone H4 (H4act) and DNA. Female genotypes are indicated. In *asf1* KD eggs, limited *de novo* histone deposition is observed in the male nucleus (arrowhead). In *Hira* KD or *yem* KD eggs, histones are barely detectable in the male nucleus. Scale bar: 10 μm. **b** Metaphase of the first zygotic mitosis. In control embryos (left), paternal chromosomes (arrowhead) stain brightly with anti-H4act antibodies and appear greenish in the merged image. In *asf1* KD embryos, the male nucleus (arrowhead) fails to condense into mitotic chromosomes and stains weakly for H4act. Scale bar: 10 μm. **c** Anaphase of the first zygotic mitosis in control (left) and *asf1* KD embryos (right). In the control, paternal chromosomes are still distinguished by the anti-H4act staining and appear yellowish. In *asf1* KD embryos, paternal chromatin (arrowhead) lags behind migrating maternal chromosomes. Scale bar: 10 μm. **d** Second mitotic division. In *asf1* KD embryos, the male nucleus (arrowhead) typically appears partially decondensed and stains weakly with anti-H4act antibodies (orange color). In contrast, the male nucleus in *yem* KD embryos remains condensed and free of H4act (it thus appears red). Scale bar: 10 μm. **e** HIRA localizes and persists in the male nucleus in the absence of ASF1. Left: Anti-HIRA antibodies normally stain the male nucleus in *asf1* KD egg during female meiosis II. Right: HIRA remains abundant in the male nucleus in *asf1* KD eggs during pronuclear apposition and the first zygotic mitosis. HIRA was detected in all eggs or cycle 1 embryos analyzed (100%; *n* = 15). Color panels show merged stainings. Scale bar: 10 μm

We made the intriguing observation that developing *asf1* KD embryos appeared haploid (Fig. 4a). At cellularization, *asf1* KD embryos showed increased nuclear density at the peripheral layer typical of haploid embryos [34] (Fig. 4b). Furthermore, KD embryos did not express a paternally transmitted *GFP*-*Cid* transgene, which encodes a fluorescent version of the centromeric histone H3 CID [35], thus indicating the absence of paternal chromosomes (Additional file 4: Fig. S3). We thus examined the fate of paternal chromosomes immediately following fertilization. In *Drosophila*, sperm DNA is almost entirely packaged with protamine-like, SNBPs [36]. Immediately following the delivery of the sperm nucleus into the egg cytoplasm, SNBPs are replaced with maternally provided histones and the paternal nucleus begins to decondense [37]. By the time of pronuclear apposition in wild-type eggs, both male and female pronuclei are similar in size and almost indistinguishable. In striking contrast, in all *asf1* KD eggs observed at the apposition stage (*n* = 46), the male pronucleus appeared abnormally small and condensed (Fig. 4c). This highly specific defect was reminiscent of the maternal effect phenotype associated with mutations in the *Hira* or *yemanuclei*n (*yem*) genes, which encode the core subunits of the HIRA complex [28, 38]. We thus analyzed de novo assembly of paternal chromatin in *asf1* KD eggs using anti-acetylated H4 (H4act) antibodies [23]. In wild-type eggs, before the onset of the first S phase, anti-H4act antibodies brightly and specifically stained the male nucleus. In contrast, acetylated H4 was only weakly detected in the male nucleus of *asf1* KD eggs, thus confirming the defective paternal chromatin assembly (Fig. 5a). The limited but consistently observed H4act staining in the male nucleus of *asf1* KD eggs contrasted with *Hira* KD or *yem* KD where paternal chromatin assembly was almost completely abolished (Fig. 5a,d). In addition, paternal chromatin in *asf1* KD eggs remained closely associated with maternal chromosomes and was thus more prone to interfere with the first division (Fig. 5b,c) compared to the inert, almost histone-free male nucleus typically observed in the absence of HIRA or YEM [28, 39]. Along the same line, *asf1* KD females produced 56.6% (*n* = 258) of embryos that never reached the blastoderm stage whereas *Hira* KD or *yem* KD females produced only 5.7% (*n* = 579) and 10.8% (*n* = 443) early arrested embryos, respectively (Fig. 3c and Additional file 3: Table S1). Nonetheless, the high proportion of early arrest *asf1* KD embryos remained essentially unchanged when *asf1* KD females were also homozygous for *Hira*^*ssm*^, a strong loss of function mutation in the *Hira* gene [23] (Fig. 3c and Additional file 3: Table S1). This confirms that *asf1* KD embryos, beside their pronuclear phenotype and haploid development, have additional defects during the syncytial phase of embryogenesis. Accordingly, the nuclear layer at the surface of *asf1* KD blastoderm embryos typically appeared irregular, with multiple patches of nuclear fallout, a phenotype indicative of the presence of damaged nuclei (Fig. 4b). Interestingly, when the *asf1* shRNA was induced in females heterozygous for a null allele of the *Caf1 p180* gene (+*/p180*^*3*^*; GL00171/nos*-*Gal4*), which encodes the large subunit of the *Drosophila* CAF-1 complex, the proportion of early arrested embryos increased to 73.6%, compared to 48.3% for control +*/FM7c; GL00171/nos*-*Gal4* females (Fig. 3c and Additional file 3: Table S1**)**. This genetic interaction between *asf1* and *Caf1 p180*, which was previously reported in another context [40], thus supports a non-essential but significant role of ASF1 during RC chromatin assembly in cleavage nuclei, likely by controlling the supply of H3 and H4 to the CAF-1 complex at replication forks.

### ASF1 is not directly involved in paternal chromatin assembly

The implication of ASF1 in paternal chromatin assembly was not anticipated since we previously reported that ASF1 was absent from the decondensing male nucleus undergoing chromatin remodeling [27]. To confirm this result, originally obtained using the anti-ASF1 antibody, we stained eggs from *g*-*asf1*-*V5* transgenic females with an anti-V5 antibody. Although the anti-V5 staining was generally weaker than the anti-ASF1 staining in early embryos, we observed in most cases (83%; *n* = 12) the expected presence of ASF1-V5 in both pronuclei shortly before apposition, at the onset of DNA replication. However, ASF1-V5 was never detected in the decondensing male pronucleus observed during meiosis II (*n *= 13), thus confirming that ASF1 does not reside in the male nucleus during RI nucleosome assembly (Additional file 5: Fig. S4). We then wondered whether the distribution of HIRA itself was affected in *asf1* KD eggs. In wild-type eggs, HIRA specifically accumulates in the decondensing nucleus where it persists until pronuclear apposition [23, 27]. We observed that, despite the observed defective chromatin assembly, HIRA localized normally in the male nucleus in *asf1* KD eggs. Furthermore, HIRA remained strongly enriched in the condensed male nucleus, even after the onset of the first mitosis (Fig. 5e). Our results thus strongly suggest that ASF1 is critically required to hand over histones to the HIRA complex prior to its translocation to the male pronucleus, while ASF1 itself does not reside on chromatin during the assembly process.

### The HIRA B domain is not essential for paternal chromatin assembly

**Fig. 6:**
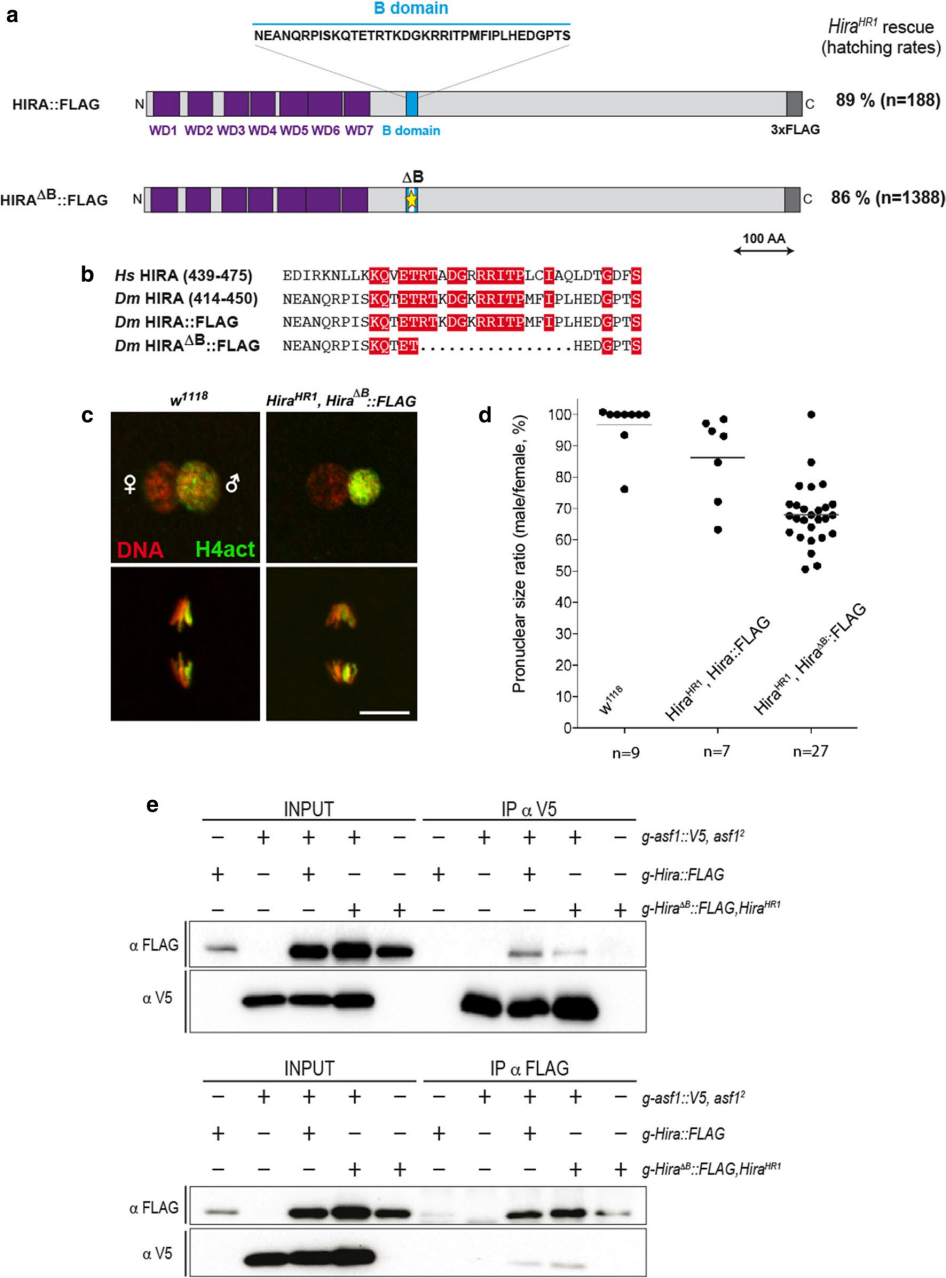
The HIRA B domain is dispensable for sperm chromatin remodeling. **a** Representation of HIRA::Flag proteins expressed from transgenes used in c-e. The position of WD40 repeats and B domain are shown. The ability of each corresponding transgene to rescue the fertility of homozygous *Hira*^*HR1*^ females (embryo hatching rate) is indicated. **b** Alignment of human (*Hs*) or *Drosophila* (*Dm*) HIRA B domains. Conserved residues are highlighted in red. The deleted residues in HIRA[ΔB]-Flag are indicated. **c** Representative images (merged DNA and H4act stainings) of pronuclear apposition (upper panels) and first zygotic anaphase (lower panels) from the indicated females. Scale bar: 10 μm. **d** Quantification of pronuclear size at apposition for the indicated genotypes are represented as a scatter plot. Horizontal lines represent means. The number of pronuclei analyzed is shown under the graph. In *Hira*^*HR1*^*, Hira*^*∆B*^*::Flag* flies, the size of male pronucleus relative to the female counterpart is significantly smaller than in control flies (Unpaired *t* test: *p* < 0.0001 *Hira*^*∆B*^*::Flag* versus wild type and p = 0.0004 *Hira*^*∆B*^*::Flag* versus *Hira::Flag*). **e** Co-immunoprecipitation experiments performed with anti-FLAG (HIRA) and anti-V5 (ASF1) on ovarian extracts from females carrying the indicated transgenes and mutant alleles. Deletion of the HIRA B domain does not abolish the ASF1-HIRA interaction in ovaries

Both structural and functional analyses have collectively established that ASF1 provides H3.3–H4 dimers to HIRA through a direct physical interaction involving a small, evolutionary conserved domain of 37 amino-acids called the HIRA B domain [13, 19, 42–44]. To address the functional contribution of this domain during paternal chromatin assembly, we generated flies expressing a HIRA::Flag protein bearing a small deletion in the B domain (*Hira[ΔB]*-*Flag*) (Fig. 6a,b). The HIRA[ΔB]::Flag recombinant protein lacks 16 residues (428-443) that correspond to the minimal peptide required for ASF1 binding [43]. Surprisingly, the *Hira[ΔB]*-*Flag* transgene fully rescued the fertility of females homozygous for a null allele of *Hira* (Fig. 6a). Cytological examination of rescued eggs confirmed that the deleted region is dispensable for paternal chromatin assembly. We noticed, however, that the male nucleus in rescued *Hira[ΔB]*-*Flag* eggs observed at pronuclear apposition appeared on average about 30% smaller in diameter than its female counterpart (Fig. 6c,d), suggesting that the rate of paternal chromatin assembly and nuclear decondensation were suboptimal in this context. To evaluate the impact of the *ΔB* deletion on the ability of HIRA to interact with ASF1 in vivo, we performed co-immunoprecipitation experiments using ovarian extracts. Interestingly, the *ΔB* deletion did not affect the HIRA-ASF1 interaction to appreciable levels (Fig. 6e). Our results thus suggest that these proteins can rely on additional or different domains for their interaction in the peculiar context of the female germline.

## Discussion

### ASF1 contributes to the maintenance of chromatin integrity during cleavage divisions

In contrast to HIRA and YEM, the two known core HIRA complex proteins in *Drosophila*, zygotic expression of ASF1 is essential for development and viability. Its well-established role in S phase progression [8, 17, 45] is indeed likely critical in *Drosophila* proliferating cells, as illustrated in this work by the dramatic impact of *asf1* KD on wing development. Unexpectedly, the depletion of ASF1 to undetectable levels in early embryos did not abolish cleavage nuclear divisions. As a matter of comparison, knocking down a catalytic subunit of the DNA polymerase epsilon complex completely blocks development as early as the first zygotic mitosis [46]. Thus, despite the abundance of ASF1 in replicating embryo nuclei, it is not absolutely required for nuclear divisions. We thus speculate that, in the highly peculiar context of the early *Drosophila* embryo, the absence of ASF1 is at least partially compensated by other histone chaperones. Interestingly, ASF1 appears equally dispensable for RC chromatin assembly in *Xenopus* egg extracts, where other H3–H4 histone chaperones, such as N1/N2, could possibly provide replicative histones to the CAF-1 complex [47]. In addition, MCM2, a conserved subunit of the minichromosome maintenance helicase, can associate with both parental and newly synthesized histones H3 and H4, opening the possibility that it supplies H3–H4 dimers to the CAF-1 complex at replication forks [17, 18, 48]. In human cells, the histone acceptor property of ASF1 has also been proposed to facilitate the unwinding of parental chromatin ahead of the replication fork [17]. In any case, our study suggests that the cooperation of ASF1 with the CAF-1 complex in early *Drosophila* embryos, although not absolutely essential, is nevertheless important for the maintenance of chromatin integrity during the rapid succession of cleavage divisions.

### ASF1 is essential for the decondensation of the male pronucleus

In this study, we have established that *Drosophila* ASF1 is critically required for histone deposition in the male pronucleus. Although de novo assembly of paternal chromatin was generally initiated in *asf1* KD eggs, the male pronucleus only faintly stained with anti-histone antibodies and nuclear decondensation was severely impaired. We have previously reported that in eggs from *yem* mutant females that were partially rescued with a transgene expressing YEM at suboptimal levels, the male pronucleus incorporated at least some histones but failed to decondense normally [28]. Similarly, in *asf1* KD eggs, the HIRA complex is still capable of limited nucleosome assembly in the male pronucleus, but this residual activity is not sufficient to ensure the timely assembly of paternal chromatin and pronuclear decondensation. Interestingly, it has been recently shown that depletion of ASF1 from *Xenopus* egg extracts prevents de novo nucleosome assembly on prepared mouse sperm nuclei [49]. The results obtained with this artificial system suggest that ASF1 could play a conserved function in assisting the HIRA complex during paternal chromatin assembly.

Importantly, we have also confirmed that ASF1 is not detectable in the male nucleus, despite its presence later on in replicating pronuclei. This paradoxical situation indicates that although ASF1 is required for male pronuclear formation, it does not directly participate in the proper assembly of H3.3-containing nucleosomes on sperm DNA. The apparently normal distribution of HIRA in the decondensing sperm nucleus of *asf1* KD eggs also indicates that the phenotype is not caused by the incapacity of the HIRA complex to enter the male pronucleus. We instead favor a model where ASF1 is required to load the HIRA core complex with H3.3–H4 dimers before its translocation to the fertilizing sperm nucleus. In the absence of maternal ASF1, preloading of the HIRA complex with H3.3 and H4 is most likely impaired, which prevents efficient assembly of paternal chromatin. This situation is reminiscent of the male pronuclear phenotype observed in eggs from *Hira*^*ssm*^ females, where the HIRA protein with a R225K substitution localizes normally to the male nucleus but fails to assemble chromatin [23]. Similarly, depletion of H3.3 in mouse zygotes impairs paternal chromatin assembly but does not prevent HIRA to localize to the paternal pronucleus [24].

### Distinct functions of ASF1 and HIRA at fertilization

Our demonstration that ASF1 is not directly involved in histone deposition in the male pronucleus also corroborates with previous findings. For instance, in H3.3 depleted salivary glands, although HIRA, XNP and ASF1 are all initially recruited at induced *Hsp70* genes, only HIRA and XNP persist on chromatin after heat-shock, suggesting that ASF1 does not reside on DNA during transcription-coupled nucleosome assembly [20]. In addition, it has been proposed that in human cells, the HIRA core complex (HIRA, UBN1 and Cabin1) fills nucleosome gaps by directly interacting with naked DNA, a property not shared by ASF1a [41]. Finally, HIRA, UBN1 and Cabin1, but not Asf1a and Asf1b, are recruited at sites of DNA damage to restore chromatin after DNA repair [50].

The assembly of paternal chromatin provides yet another dramatic illustration of the different properties of ASF1 and HIRA complex proteins with respect to histone deposition on DNA. Our study strongly indicates that the handover of H3.3–H4 dimers from ASF1 to the HIRA complex is indeed spatially and temporally uncoupled from the proper nucleosome assembly process. Once loaded with histones, the HIRA complex must reach the sperm nucleus and target sperm DNA regions that become exposed following SNBP eviction. This likely involves the DNA binding property of the HIRA/YEM complex [28, 51] in a way similar to the nucleosome gap filling mechanism already mentioned. Assembly of paternal chromatin is, however, unique as it occurs at the scale of the whole male nucleus and takes place in the very large volume of the egg cell. The HIRA complex preloading with H3.3–H4 dimers could facilitate the rapid and efficient assembly of paternal chromatin, which must take place within minutes after the delivery of the sperm nucleus in the egg cytoplasm.

How the partitioning of H3.2–H4 and H3.3–H4 histone dimers on ASF1 is established before zygote formation remains, however, poorly understood. A recent study has proposed that the higher abundance of ASF1 in H3.3 complexes purified from early *Drosophila* embryos could indicate a higher affinity of ASF1 for H3.3 over H3.2 [52]. If true, this difference could fit well with the role of ASF1 in providing H3.3–H4 dimers to the HIRA complex at fertilization. In *Drosophila* eggs, the absence of ASF1 in the male pronucleus at least indicates that HIRA and ASF1 no longer interact during nucleosome assembly. Interestingly, it has been recently shown that the specificity of the human HIRA complex for H3.3–H4 over H3.1–H4 is conferred by UBN1, and not by HIRA itself [53]. The UBN1-H3.3 binding involved specific residues of the UBN1 HRD domain, which are conserved in the *Drosophila* orthologous protein YEM [53]. In *Drosophila*, ASF1 has been previously shown to interact with YEM in co-immunoprecipitation experiments [54]. We thus speculate that YEM, through its interaction with ASF1-H3.3–H4 complexes, controls the specific handover of H3.3–H4 over H3.2–H4 to the HIRA complex in the cytoplasm of *Drosophila* eggs. In this context, the HIRA B domain could play a secondary role, perhaps by reinforcing this interaction.

## Conclusions

Our functional characterization of ASF1 has established the role of this histone chaperone in the maintenance of chromatin integrity during female meiosis and the amplification of embryonic nuclei. This work also revealed that ASF1 is critically required to assist the HIRA complex during the assembly of paternal chromatin. ASF1 provides H3.3–H4 histone dimers to the HIRA complex but itself does not reside in the male pronucleus during de novo chromatin assembly. The spatio-temporal uncoupling of histone loading on the HIRA complex and histone deposition on paternal DNA illustrates the adaptability of histone chaperones and chromatin assembly factors to control such a highly constrained chromatin assembly process. Further work should aim at understanding how this essential cooperation is orchestrated during egg activation and fertilization.

## Methods

### *Drosophila* stocks

Flies were raised at 25 °C (unless otherwise indicated) on standard cornmeal-agar-yeast medium. The *w*^*1118*^ strain was used as a wild-type control. The following stocks were obtained from the Bloomington Drosophila Stock Center (simplified genotypes are given): *asf1*^*1*^(#9547) [30], *asf1*^*2*^ (#9548) [30], *P{TRiP.GL00349}attP2* (*yem* shRNA; #35425), *P{TRiP. GL00257}attP2* (*Hira* shRNA; #35346), *P{TRiP.HMC03937}attP40* (*asf1* shRNA; #55250), *P{TRiP. GL00171}attP2* (*asf1* shRNA; #35273), *P{GAL4*-*vg.M}2* (#6819; *vg*-*Gal4*), *P{Act5C*-*GAL4}*^*25FO1*^ (#4414), *P{otu*-*GAL4::VP16.R}1; P{GAL4*-*nos.NGT}40; P{GAL4::VP16*-*nos.UTR}MVD1* (Maternal Triple Driver or “MTD-Gal4”; #31777), *P{GAL4::VP16*-*nos.UTR}MVD1* (“nos-Gal4”; #4937). Other stocks are: *w*^*1118*^ *Hira*^*ssm*^*/FM7c* [55], *w*^*1118*^ *Hira*^*HR1*^*/FM7* *h* [27], *P[w *+ *, g*-*EGFP*-*cid]III.2* [35], *p180*^*3*^*/FM7i* [40], *P[w *+ *, Hira::3xFlag]II/CyO* [23] and *P[w *+ *, HiraΔB::3xFlag]I/FM7* (this study).

### Transgenic constructs

#### P[w + , HiraΔB::3xFlag]

The plasmid *P[w *+ *, Hira::3xFlag]* [23] was used as a template to amplify a 2440-bp fragment from -400 upstream the ATG to +2040 by PCR using primers HIRA*Bgl*II (5′-CGCCCGCGGAAAGATCTATTCTTATATG-3′) and HIRAPEPT13′ (5′-TGGATCCGCGCAATGCACTGCAGAACT-3′). The PCR fragment was cloned into the PGEM-T vector (Promega) and verified by sequencing. The resulting plasmid was used as a template to generate a deletion in the region corresponding to the B domain minimal region [43]. Two mutagenic primers DeletBfwd (5′-AGCGACCCATTAGTAAACAAACGGAAACGCACGAAGATGGACCCACATCGCTGA-3′) and DeletBrev (5′-TCAGCGATGTGGGTCCATCTTCGTGCGTTTCCGTTTGTTTACTAATGGGTCGCT-3′) were used to delete a 48 bp region (corresponding to aa 428 to 443 of the HIRA protein) using the Quickchange II site directed mutagenesis kit (Stratagene). The construct was verified by sequencing, digested by BglII and reinserted into *P[w *+ *, Hira::3xFlag]* to generate the *P[w *+ *, HiraΔB::3xFlag].* Transgenic flies were obtained by standard P-mediated germline transformation.

#### P[w + , g-asf1::V5]attB

A 1920-bp fragment covering de 5′ regulatory sequences and the complete coding sequence (CDS) of *asf1* was amplified by PCR from *y w* genomic DNA and subcloned into the pGEM-T vector (Promega). Primers used were: 5′CGGCGGCCGCCAGTCCACATCACA3′, which introduces a *Not*I restriction site and 5′ TTGGTACCTCACGTAGAATCGAGACCGAGGAGAGGGTTAGGGATAGGCTTACCACATTCCATGGCCAGTGAGT 3′ which introduces a V5 tag, stop codon and *Kpn*I restriction site. Similarly, a 836-bp fragment covering the downstream regulatory region of *asf1* was amplified by PCR from *y w* genomic DNA and subcloned into the pGEM-T vector using the following primers: 5′GGTACCGAGCTGCCGGCAGTCCGAAC3′ and 5′TTCTAGACTGATCCGAGGGCAACTGCC3′ containing *Kpn*I and *Xba*I restriction sites, respectively. Both fragments were excised and cloned into the *pattB* vector (www.flyc31.org) using *Not*I, *Kpn*I and *Kpn*I, *Xba*I restriction sites, respectively.

#### P [w + , UASP-asf1::V5]attB

The *asf1* full-length coding sequences was amplified by PCR from *y w* genomic DNA using the following primers: 5′GAGGTACCATGGCCAAGGTGCACATCAC3′ and 5′TTGGTACCTCACGTAGAATCGAGACCGAGGAGAGGGTTAGGGATAGGCTTACCACATTCCATGGCCAGTGAGT3′. Both primers include a *Kpn*I restriction site subsequently used for cloning into the *pUASP*-*attB vector* (www.flyc31.org).

All *asf1::V5* transgenes were integrated in the *PBac{attP*-*3B}VK00031* platform (62E1) using PhiC31-mediated transformation [56].

### Fertility tests

To obtain KD females, virgin shRNA transgenic females were mass crossed with transgenic Gal4 males at 25 °C and females of the desired genotype were recovered in the F1 progeny. To measure fertility, virgin females were aged for 3 days at 25 °C in the presence of males and were then allowed to lay eggs on standard medium for 24 h. Embryos were counted and then let to develop at least 36 h at 25 °C. Embryo hatching rates were then calculated.

### Protein immunoprecipitation and Western blotting

About 50 µl of adult ovaries were dissected and homogenized in 2 volumes of lysis buffer [20 mM Hepes pH 7.9, 100 mM KCl, 0.1 mM EDTA, 0.1 mM EGTA, 5% Glycerol, 0.05% Igepal (Sigma Aldrich #9002-93-1) with a protease inhibitor cocktail (Roche #04693159001). Protein extracts were cleared by centrifugation and stored at − 80 °C.

Embryos were collected every 60 min and dechorionated in bleach. Protein extracts were prepared from approximately 20 µl of embryos, as described for gonads.

For co-immunoprecipitation experiments, protein extracts were prepared from ovaries of females expressing ASF1 and/or HIRA proteins fused with C-terminal V5 or 3xFLAG tags, respectively. 100 µg of protein extracts were incubated with 30 µl of prewashed anti-V5 agarose beads (Sigma Aldrich #A7345) or anti-FLAG M2 affinity gel (Sigma Aldrich #A2220) overnight at 4 °C. The beads were extensively washed with washing buffer [20 mM Hepes pH 7.9, 100 mM KCl, 200 mM NaCl, 0.1 mM EDTA, 0.1 mM EGTA, 5% Glycerol, 0.05% Igepal CA-630].

Before loading, samples were boiled in Laemmli buffer containing DTT. Electrophoresis was carried out on 10% SDS polyacrylamide gel, and Western blotting was performed using the ECL prime Western blotting detection reagent and following the manufacturer’s instructions (GE Healthcare). The antibodies were: mouse monoclonal anti-α-Tubulin (Sigma Aldrich #T9026, 1:500), rabbit polyclonal anti-ASF1 (1:2000), rabbit polyclonal anti-V5 (Invitrogen #R960-25, 1:500), mouse monoclonal anti-Flag M2 (Sigma Aldrich #F3165, 1:25000), HRP-conjugated goat anti-mouse (Biorad #170-5047; 1:50 000) and peroxidase-conjugated goat anti-rabbit (ThermoScientific #32460; 1:20 000). The experiment was performed twice with similar results.

### Immunofluorescence and microscopy

Embryos were collected on grape juice-agar plates, dechorionated in bleach, fixed in a 1:1 heptane/methanol mixture and stored at − 20 °C. For immunostaining experiments, embryos were washed three times (10 min each) in PBS, 0.1% Triton X100. Embryos were then incubated with primary antibodies in the same buffer on a wheel overnight at 4 °C and washed three times (20 min each) in PBS, 0.1% Triton X100. Incubations with secondary antibodies were performed identically. For propidium iodide staining, embryos were incubated for 1 h in a 2 mg per ml RNAse A solution at 37 °C. Embryos were finally mounted in mounting medium (Dako) containing propidium iodide or DAPI.

Ovaries were dissected in PBS-Triton 0,1% and fixed at room temperature in 4% formaldehyde in PBS for 25 min. Immunofluorescence was performed as for embryos except for the secondary antibodies that were incubated four hours at room temperature. Ovaries were then stained with propidium iodide and mounted as described above.

Primary antibodies used were: mouse monoclonal anti-GFP (Roche #118144600001; 1:200), mouse monoclonal anti-V5 (Invitrogen #R960-25, 1:500), rabbit polyclonal anti-polyacetylated Histone H4 (Millipore #06-589; 1:250), mouse monoclonal anti-histones (Millipore #MABE71; 1:1000), rabbit polyclonal anti-Hira (PG1 purified, 1:500), rabbit polyclonal anti-ASF1 [30] (A gift from F. Karch; 1:1000) and mouse monoclonal anti-PCNA (Abcam #ab29, 1:1000). Secondary antibodies were used at a 1:1000 dilution and included goat anti-mouse and goat anti-rabbit antibodies conjugated to AlexaFluor (Jackson ImmunoResearch). Images were acquired on a LSM 510 META (Carl Zeiss), and the images were processed with Adobe Photoshop and Adobe Illustrator. At least two independent experiments were performed for each immunostaining.

## Additional files

**Additional file 1: Fig. S1.** *asf1* KD embryos develop without detectable levels of ASF1. Confocal images of a nuclear cycle 10 control embryo (left) and a nuclear cycle 11 *asf1* KD embryo (right) stained for DNA (red) and ASF1 (green). ASF1 is not detected in the nuclei of *asf1* KD embryo. Scale bar: 20 μm.

**Additional file 2: Fig. S2.** Nuclear defects in early *asf1* KD embryos. Confocal images of control and *asf1* KD embryos at the indicated stage stained with anti-histone antibodies. Yellow arrows indicate chromatin bridges. Red arrow indicates disintegrated nuclei. Embryos are delineated by a dashed line. PB: Polar bodies. Scale bar: 50 μm.

**Additional file 3: Table S1.** Developmental arrest of embryos.

**Additional file 4: Fig. S3.** *asf1* KD embryos lack paternal chromosomes. Late control and *asf1* KD embryos fathered by *GFP::cid* transgenic males. Zygotic expression of the paternal centromeric marker is only detected in nuclei of control embryos (arrows in left inset). n = 140 for control embryos and n = 40 for *asf1* KD embryos. Scale bar: 50 μm.

**Additional file 5: Fig. S4.** ASF1::V5 is not detected in the decondensing male pronucleus. **a:** An egg in metaphase of meiosis II from a *g*-*asf1::V5* transgenic female stained with anti-V5 antibodies. Scale bar: 50 μm. **b:** Pronuclear migration. Scale bar: 10 μm. **c:** ASF1::V5 is incorporated in both pronuclei at the onset of DNA replication. Scale bar: 10 μm. **d:** Pronuclei stained with anti-V5 antibodies during apposition. Scale bar: 10 μm.

## Abbreviations

ASF1: Anti-Silencing Factor-1
RC: Replication-Coupled
RI: Replication-Independent
CAF-1: Chromatin Assembly Factor-1
shRNA: small hairpin RNA
KD: knocked-down
HIRA: Histone Regulator-A
ATRX: alpha thalassemia/mental retardation syndrome X-linked
XNP: X-linked nuclear protein
UBN1: ubinuclein-1
SNBP: sperm nuclear basic protein
YEM: yemanuclein
UAS: upstream activating sequence
UTR: untranslated region
GV: germinal vesicle
CID: centromere identifier
MCM2: minichromosome maintenance-2
HRP: horseradish peroxidase
GFP: green fluorescent protein
PCNA: proliferating cell nuclear antigen
