# Supplementary figures and images for: ASF1 is required to load histones on the HIRA complex in preparation of paternal chromatin assembly at fertilization
Source: Epigenetics Chromatin. 2018 May 11;11:19. doi: 10.1186/s13072-018-0189-x (PMC5946387; doi:10.1186/s13072-018-0189-x)

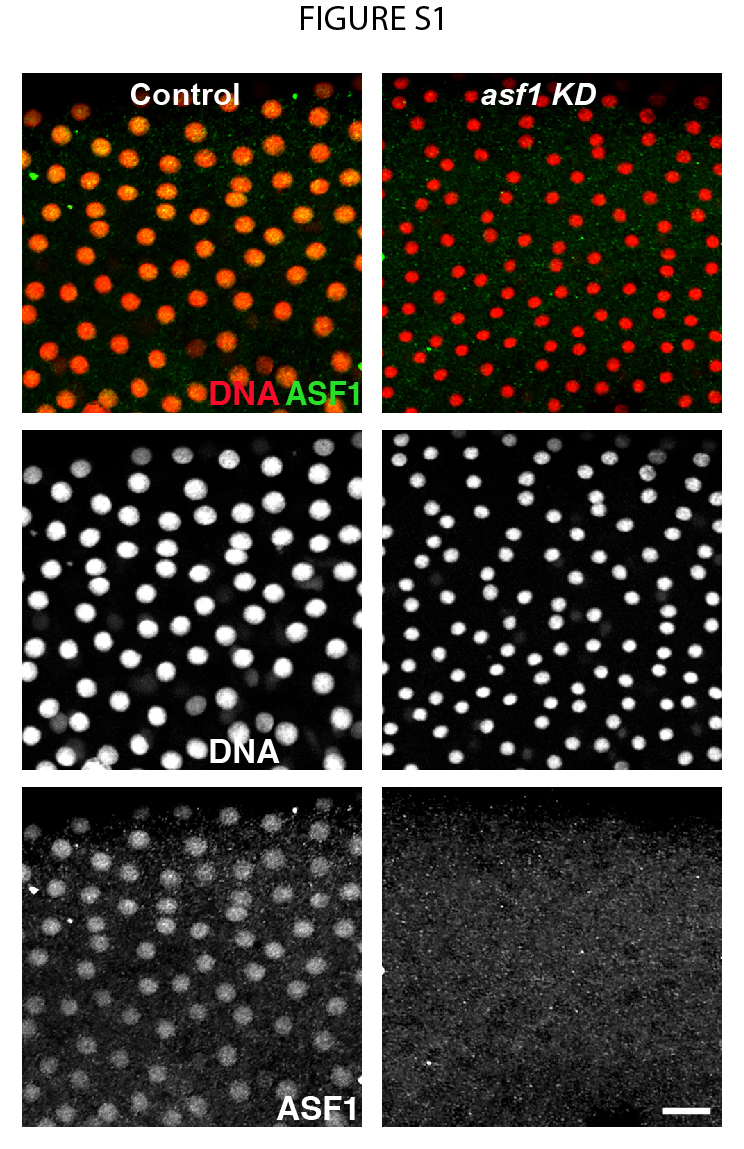

Supplement: Supplementary file 1 — Additional file 1: Fig. S1. asf1 KD embryos develop without detectable levels of ASF1. Confocal images of a nuclear cycle 10 control embryo (left) and a nuclear cycle 11 asf1 KD embryo (right) stained for DNA (red) and ASF1 (green). ASF1 is not detected in the nuclei of asf1 KD embryo. Scale bar: 20 μm. [file 13072_2018_189_MOESM1_ESM.jpg]

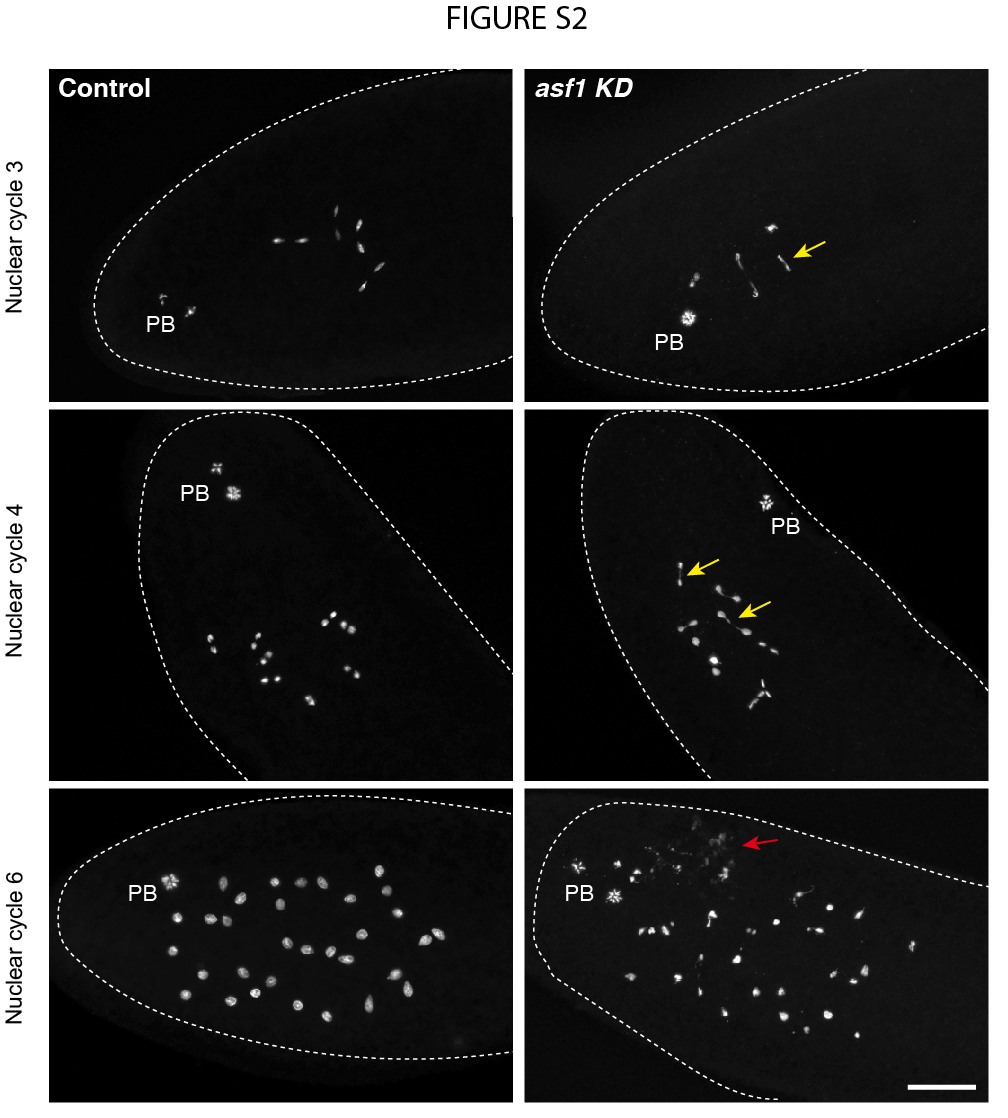

Supplement: Supplementary file 2 — Additional file 2: Fig. S2. Nuclear defects in early asf1 KD embryos. Confocal images of control and asf1 KD embryos at the indicated stage stained with anti-histone antibodies. Yellow arrows indicate chromatin bridges. Red arrow indicates disintegrated nuclei. Embryos are delineated by a dashed line. PB: Polar bodies. Scale bar: 50 μm. [file 13072_2018_189_MOESM2_ESM.jpg]

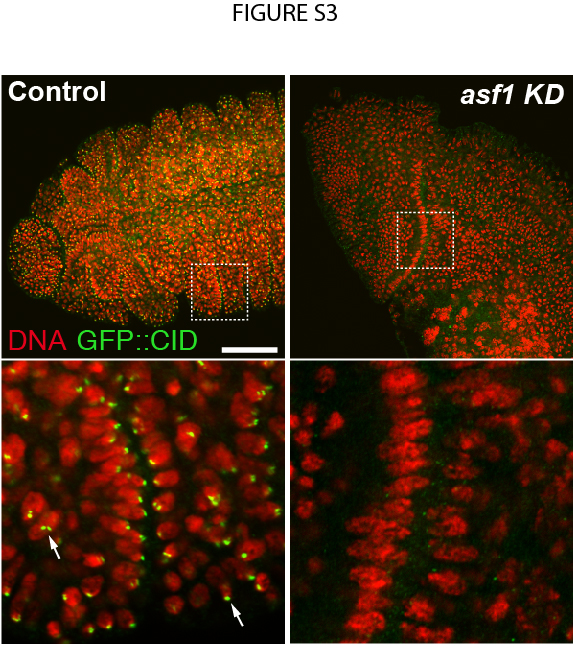

Supplement: Supplementary file 4 — Additional file 4: Fig. S3. asf1 KD embryos lack paternal chromosomes. Late control and asf1 KD embryos fathered by GFP::cid transgenic males. Zygotic expression of the paternal centromeric marker is only detected in nuclei of control embryos (arrows in left inset). n = 140 for control embryos and n = 40 for asf1 KD embryos. Scale bar: 50 μm. [file 13072_2018_189_MOESM4_ESM.jpg]

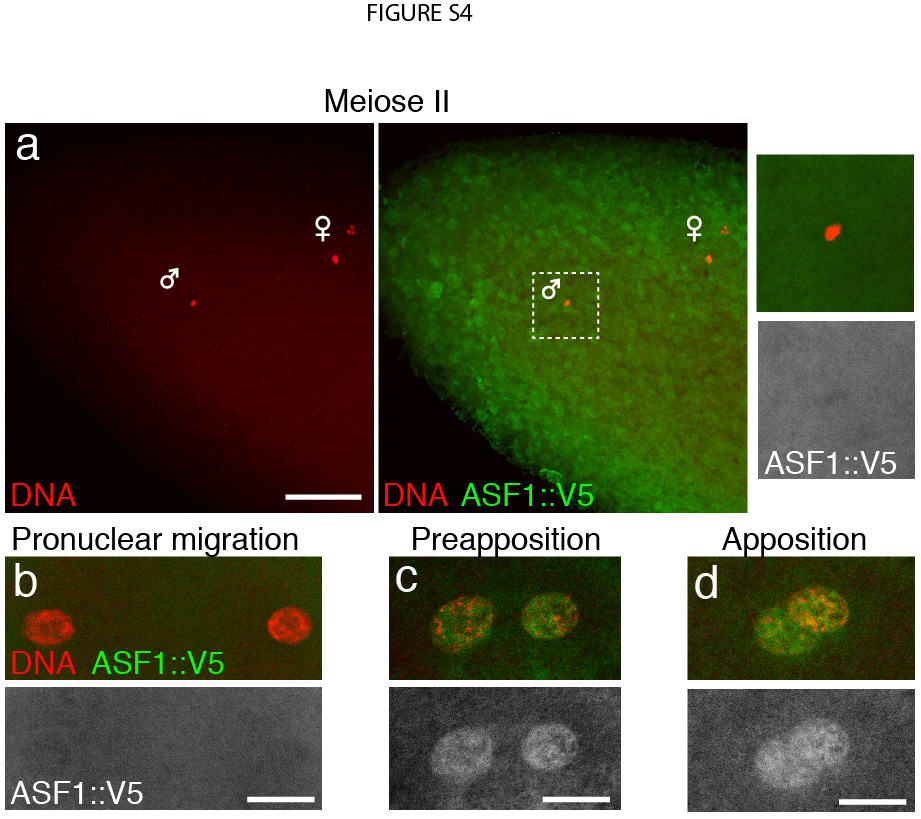

Supplement: Supplementary file 5 — Additional file 5: Fig. S4. ASF1::V5 is not detected in the decondensing male pronucleus. a: An egg in metaphase of meiosis II from a g-asf1::V5 transgenic female stained with anti-V5 antibodies. Scale bar: 50 μm. b: Pronuclear migration. Scale bar: 10 μm. c: ASF1::V5 is incorporated in both pronuclei at the onset of DNA replication. Scale bar: 10 μm. d: Pronuclei stained with anti-V5 antibodies during apposition. Scale bar: 10 μm. [file 13072_2018_189_MOESM5_ESM.jpg]
