# Supplementary material for: ASF1 is required to load histones on the HIRA complex in preparation of paternal chromatin assembly at fertilization
Source: Epigenetics Chromatin. 2018 May 11;11:19. doi: 10.1186/s13072-018-0189-x (PMC5946387; doi:10.1186/s13072-018-0189-x)
Supplement: Supplementary file 3 — Additional file 3: Table S1. Developmental arrest of embryos. [file 13072_2018_189_MOESM3_ESM.pdf]

Table S1 - Developmental arrest of embryos

**Maternal genotype**	2- 4h old embryos				
	**n**	**< cycle 9** (Early arrest)	**9 < cycle ≤14** (Blastoderm)	**≥ Gastrula**	**% early arrest**
MTD-Gal4 > UAS-shRNA asf1	258	146	97	15	56.6
MTD-Gal4 > UAS-shRNA Hira	579	33	358	188	5.7
MTD-Gal4 > UAS-shRNA yem	443	48	309	86	10.9
nos-Gal4> UAS-shRNA asf1	208	95	71	42	45.7
nos-Gal4> UAS-asf1::V5(62E), UAS-shRNA asf1	153	5	86	62	3.3
Hira[ssm]	225	45	99	81	20.0
Hira[ssm]; nos-Gal4>UAS-shRNA asf1	201	111	61	29	55.2
+/FM7c ; nos-Gal4> UAS-shRNA asf1	346	167	130	49	48.3
$p180^3$/+; nos-Gal4> UAS-shRNA asf1	182	134	37	11	73.6
w^{1118}	172	7	34	131	4.0

Note: Females were crossed either with w^{1118} or *gcid-EGFP::cid* males. Eggs were collected 2h after egg deposition and were stored at 25°C for up to 2h before fixation.
